# Modelling bacterial speciation

**DOI:** 10.1098/rstb.2006.1926

**Published:** 2006-10-06

**Authors:** William P Hanage, Brian G Spratt, Katherine M.E Turner, Christophe Fraser

**Affiliations:** Department of Infectious Disease Epidemiology, Imperial College London, St Mary's Hospital CampusNorfolk Place, London W2 1PG, UK

**Keywords:** Fisher–Wright model, simulation, species, recombination, multilocus genotypes, genetic cartography

## Abstract

A central problem in understanding bacterial speciation is how clusters of closely related strains emerge and persist in the face of recombination. We use a neutral Fisher–Wright model in which genotypes, defined by the alleles at 140 house-keeping loci, change in each generation by mutation or recombination, and examine conditions in which an initially uniform population gives rise to resolved clusters. Where recombination occurs at equal frequency between all members of the population, we observe a transition between clonal structure and sexual structure as the rate of recombination increases. In the clonal situation, clearly resolved clusters are regularly formed, break up or go extinct. In the sexual situation, the formation of distinct clusters is prevented by the cohesive force of recombination. Where the rate of recombination is a declining log-linear function of the genetic distance between the donor and recipient strain, distinct clusters emerge even with high rates of recombination. These clusters arise in the absence of selection, and have many of the properties of species, with high recombination rates and thus sexual cohesion within clusters and low rates between clusters. Distance-scaled recombination can thus lead to a population splitting into distinct genotypic clusters, a process that mimics sympatric speciation. However, empirical estimates of the relationship between sequence divergence and recombination rate indicate that the decline in recombination is an insufficiently steep function of genetic distance to generate species in nature under neutral drift, and thus that other mechanisms should be invoked to explain speciation in the presence of recombination.

## 1. Introduction

Despite the well-documented extent of lateral gene transfer among the prokaryotes (Gogarten *et al*. 2002), which has led some researchers to doubt whether the term ‘species’ has any meaning for these organisms (Lawrence 2002; Gevers *et al*. 2005), we can define clusters of similar phenotype or genotype which often correspond to named species. However, these clusters show a variety of forms, levels of within-cluster diversity and degree of resolution from neighbouring clusters. This should not be surprising, because bacteria may have very different lifestyles and rates of recombination (Hanage *et al*. 2006*a*). Surprisingly few studies have addressed the necessary conditions for the formation of genotypic clusters, their dynamics or properties, which could shed light on the reasons why, even in the face of promiscuous recombination, we can define entities among the bacteria which we recognize as species (Hanage *et al*. 2005*a*,*b*).

In recent years, the ability of the sequences of multiple house-keeping genes to identify distinct genotypic clusters among populations of closely related species has been explored. This approach (reviewed elsewhere in this volume; Hanage *et al*. 2006*b*) is predicated upon the success of multilocus sequence typing (MLST) for both precise strain characterization in the context of epidemiology (Hanage *et al*. 2004) and application to species definition termed ‘multilocus sequence analysis’ (MLSA; Gevers *et al*. 2005). In MLSA, the sequences of several (usually about seven) house-keeping genes are determined, and the concatenates of these are used to determine the presence (or otherwise) of clusters of related genotypes in sequence space, which may be related to existing species or used to inform the process of species assignment. This approach has demonstrated that at least some of the species presently recognized by microbiologists are concordant with genotypic clusters, even when a large number of strains of frequently recombining species are considered (Hanage *et al*. 2005*a*,*b*).

A complementary approach is to identify by simulation the conditions under which an initially uniform bacterial population diverges and resolves into distinct genotypic clusters. While much of the previous work in this field has concentrated on the role of periodic sweeps in ecologically structured populations in the formation of distinct genotypic clusters (ecotypes; Majewski & Cohan 1999*a*; Cohan 2002), we deliberately explore the behaviour of systems under neutral drift. Neutrality provides a useful null model and identifies conditions under which genotypes do, or do not, form resolved clusters in the absence of selection or population subdivision (Fraser *et al*. 2005). In addition, neutrality is not necessarily a statement of lack of selection or structure, but rather an effective description of situations where the evolutionary landscape is highly complex, and thus specific genotypes are rarely advantageous over extended periods of time or space. The effect of more persistent selection or population substructure in affecting basic neutral speciation models can subsequently be explored.

## 2. Simulating populations using high-resolution multilocus sequence typing

In MLST, internal fragments (approx. 500 bp) of seven house-keeping genes are sequenced from each strain (Maiden *et al*. 1998). The different sequences at each locus are assigned different allele numbers and each strain is defined by a string of seven integers (the allelic profile), corresponding to the alleles at seven loci. Large MLST datasets are available for several bacterial species, and we have described a population genetic model that can be applied to this rich source of data (Fraser *et al*. 2005; Hanage *et al*. 2006*a*).

This Fisher–Wright neutral model of bacterial evolution defines the genotypes of strains in the same way as MLST, by the alleles present at seven housekeeping loci. Multilocus genotypes in generation *n*+1 of the neutral model are randomly sampled from generation *n*, with individual loci changing at specified rates by point mutation or recombination. The analytical solution for the expected distribution of allelic mismatches of multilocus genotypes at equilibrium for any mutation and recombination rate allows these model parameters to be estimated from samples of real bacterial populations characterized by MLST (Fraser *et al*. 2005). We may also simulate bacterial populations for any values of the mutation and recombination rates. To explore speciation, we have increased the number of loci that define each strain from 7 to 140, as this provides a greatly enhanced ability to discriminate between strains. Our choice for the number of loci as well as an allelic structure rather than full genotype simulations were motivated by the goal of substantially extending models in terms of population size and discrimination between genotypes, and were bounded by computational limitations. Simulating the evolution of populations with strains defined by large allelic profiles rather than full sequences for a limited numbers of alleles was hypothesized to limit the distorting effects of recombination in assessing genetic distance between different strains.

We simulate populations of constant size (10^6^), with each strain defined as a string of integers corresponding to the alleles at 140 loci (each of 500 bp) distributed around the chromosome. In each generation of the model, loci mutate with probability *m* and recombine with probability *r*, as previously described (Fraser *et al*. 2005). We assume an infinite alleles model in which each mutation generates an allele not previously recorded in the population. In the limiting case of full panmixis, we model recombination as replacing the allele at a single locus with another drawn at random from the population. We consider recombination to be equally probable at any of the 140 loci. By analogy with *θ* (the population mutation rate), we define the population recombination rate *ρ*=2*rN*.

In order to simulate the effect of sequence divergence on the probability of successful recombination (distance-scaled recombination), we use the allelic distance between the strain that donates the allele and the recipient strain (the proportion of allelic differences at the 140 loci) as a proxy for the sequence divergence between the strains. The probability of successful recombination in the distance-scaled recombination model declines in a log-linear fashion with increasing divergence between donor and recipient strains, as found to be the case in *Bacillus subtilis* (Majewski & Cohan 1999*b*), *Escherichia coli* (Vulic *et al*. 1997) and *Streptococcus pneumoniae* (Majewski *et al*. 2000). While in these cases the degree of local divergence between donor and recipient sequences determines the probability of successful recombination, owing to the high level of discrimination offered by the large number of loci studied we argue that our model based on mismatches in allelic profiles captures this relationship in a probabilistic sense.

Starting with an initially uniform population of 10^6^, we allow the simulation to run. At intervals, samples of 1000 strains are drawn at random and are used to examine the pattern of genotypic clustering. To display the clustering of genotypes in the samples of the simulated populations (the genetic cartography), we use a multidimensional scaling (MDS) algorithm implemented in R (Venables & Ripley 2002; Team 2005), which represents in two dimensions the overall genetic distance between each strain and all others in the population.

## 3. Clonal populations and the influence of increasing recombination

The relative contributions of point mutation and recombination to the divergence of multilocus genotypes differ greatly among bacterial populations (Feil & Spratt 2001). In some species, such as *Mycobacterium tuberculosis*, convincing evidence for recombination is lacking and genotypes diverge exclusively (or nearly so) by mutation (Smith *et al*. 2003). At the other extreme, in some populations, alleles at house-keeping loci change much more frequently by recombination than point mutation (Spratt *et al*. 2001; Hanage *et al*. 2006*a*). Therefore, we explore the patterns of clustering observed within simulated populations evolving with a fixed population mutation rate (*θ*=2) and a range of population recombination rates, from *ρ*=0 (clonal) to 20. In the initial simulations, recombination occurs at the same defined rate between any pair of strains. We then introduce the more plausible scenario of distance-scaled recombination, incorporating the log-linear reduction in recombination rate with increasing divergence between strains.

**Figure 1:**
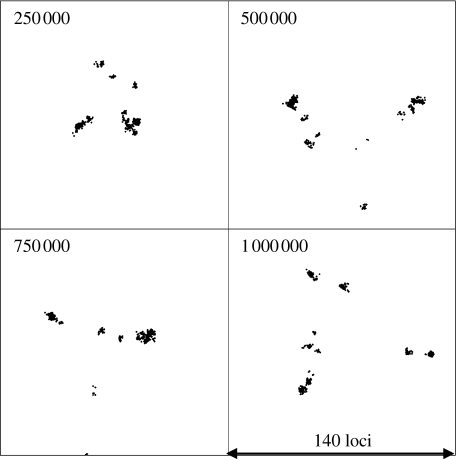
Genetic cartography of samples taken during the evolution of a population in the absence of recombination. Samples of 1000 were drawn at intervals from an evolving population of 10^6^ bacteria with *θ*=2. The relationships between these strains were measured by the pairwise allelic mismatches at 140 loci and were displayed by MDS. The number of generations of the simulation is shown in the top left of each panel. All strains are initially identical.

**Figure 2:**
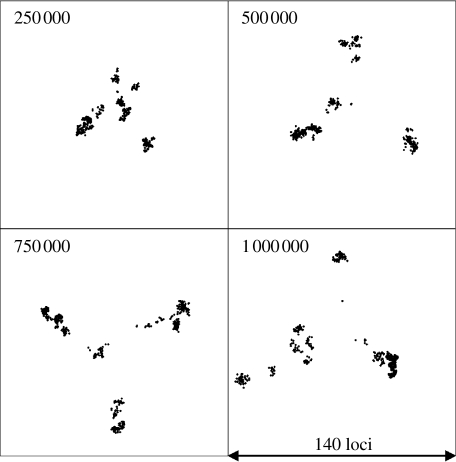
Genetic cartography of samples taken during the evolution of a population with a low rate of recombination. Details are as in figure 1, except that recombination occurs with the same frequency as mutation (*θ*=2, *ρ*=2).

**Figure 3:**
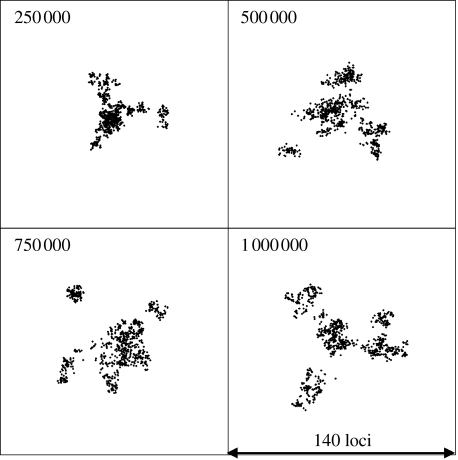
Genetic cartography of samples taken during the evolution of a population with high rates of recombination. Details are as in previous figures, except *θ*=2 and *ρ*=20.

Figure 1 shows the clusters obtained using MDS for population samples drawn every 2.5×10^5^ generations for a clonal population with *θ*=2. The diversification of the initially uniform population in the absence of recombination leads within about 250 000 generations to distinct clusters of closely related strains. The initial cluster persists, but through stochastic drift it has become extinct by the 800 000th generation. If we introduce recombination at the same rate as mutation (*θ*=2, *ρ*=2), then diversification proceeds as under the totally clonal simulation, with multiple distinct clusters emerging (figure 2). Under conditions of much higher recombination rates (*θ*=2, *ρ*=20; figure 3), transient diffuse clusters of strains still emerge, but fail to become established as distinct resolved clusters, being drawn back into the main cluster by recombination with the other strains in the population. The dynamics of clonal clustering and the cohesive effects of recombination can be observed more clearly in the movies in the electronic supplementary material.

## 4. The effect of distance-scaled recombination

**Figure 4:**
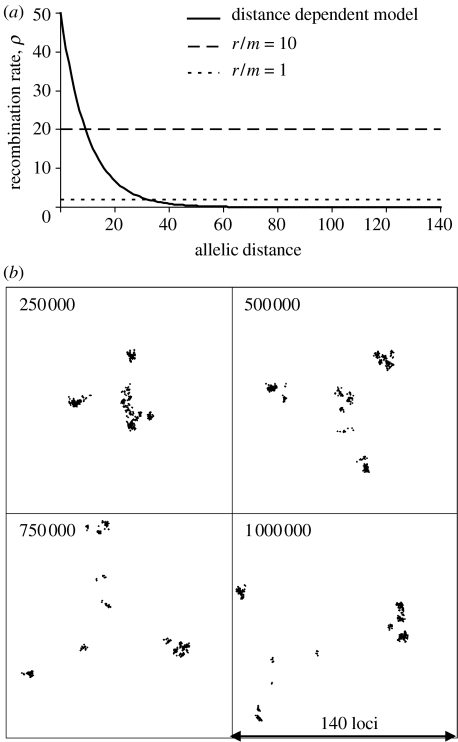
Evolution of a population with distance-scaled recombination. (*a*) The declining rate of recombination with increasing genetic (allelic) distance between strains is shown by the solid line. The dashed line shows the situation in figures 2 and 3, in which recombination is equally probable between all strains. Allelic distance is the number of the 140 loci that differ between the donor and recipient strain. (*b*) Genetic cartography of samples taken during the evolution of a population with distance-scaled recombination. *θ*=2 and *ρ*=50 for recombination between identical strains and *ρ* declines as a log-linear function of the genetic distance between strains, as described in the text.

Figure 4 shows the effect of introducing a log-linear decline in the probability of recombination with increasing overall allelic distance. For all strains *θ*=2, and the probability of recombination occurring between identical strains is very high (*ρ*_0_=50). The probability of recombination occurring between strains with allelic distance *D* is given by *ρ*=*ρ*_0_exp[−*αD*] and the resulting relationship between *ρ* and allelic distance is shown in figure 4*a*, corresponding to *α*=0.1. As shown in figure 4*b* (and a movie in the electronic supplementary material), the initially uniform cluster rapidly forms distinct clusters, which then continue to diversify and form new clusters by the same process. Between the similar strains within these distinct clusters, the rate of recombination is high (figure 4*a*). Between strains in different clusters it is low, owing to the log-linear drop-off of recombination with genetic distance. The integrity of clusters is therefore maintained by frequent recombination. Major new clusters are formed rarely, as stochastic drift infrequently leads to the establishment of new strains that are sufficiently distant from the progenitor cluster that they can no longer be reabsorbed by recombination. The number of such clusters presumably increases monotonically as a function of *θ*.

## 5. Dynamics of major cluster formation

**Figure 5:**
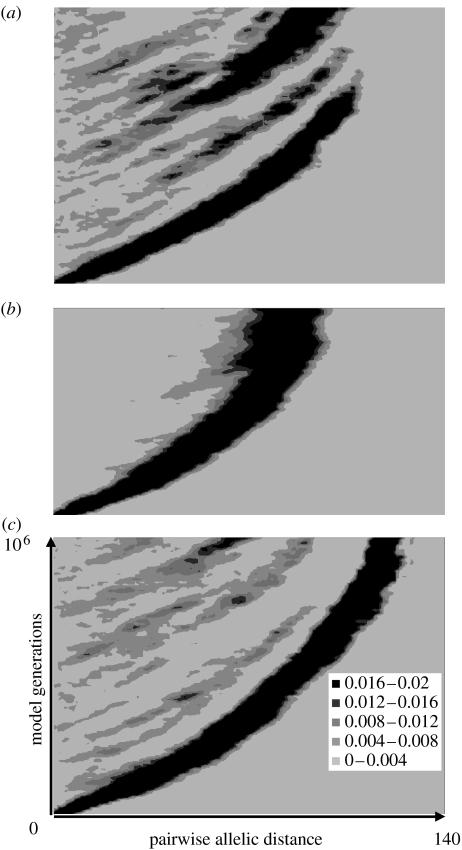
Dynamics of major cluster formation, divergence and extinction. Pairwise allelic distance is shown on the *x*-axis and time (in model generations) on the *y*-axis. The proportion of the population within each area of the figure is shown by shading according to the scale shown. Clusters of similar strains are visible as shaded areas close to the *y*-axis and their diversification is represented by an increase in genetic (allelic) distance with time. The composition of the population at any generation of the model can be seen by drawing a horizontal line at that position. (*a*) Representation of the population sampled in figure 1. Numerous small clusters emerge and become extinct. (*b*) The population sampled in figure 3. A single large cluster persists and becomes more divergent over the timespan studied. (*c*) The population sampled in figure 4*b*, in which there is distance-scaled recombination and multiple clusters arise.

Figure 5 shows an alternative way of displaying the formation and dynamics of the emergence and persistence over time of genotypic clusters under the scenarios described previously. In this representation, the change over time in the proportion of pairs of strains differing at *n* of 140 loci is shown for the entire population of 10^6^ (as opposed to samples of 1000). This analysis is unable to detect the minor transient clusters visible in the MDS diagrams, but instead focuses on the major lineages of similar strains. Figure 5*a* displays the development of the clonal population shown in figure 1. The initial cluster fragments rapidly and becomes extinct before the end of the simulation. However, by then, the population is mainly composed of a daughter cluster, which is marginally less diverse. With high rates of recombination (*θ*=2, *ρ*=20), clear clusters fail to form, and the resulting population is instead highly diverse and diffuse (shown by the broad band in figure 5*b*, indicating that most strains share only around half their alleles with most others in the population). The effect of distance-scaled recombination (figure 5*c*) is clearly visible in the multiple resolved clusters that emerge.

## 6. Discussion

While any satisfying definition of bacterial species remains elusive, the present work demonstrates that distinct clusters of similar genotypes can emerge under many parameter values for both mutation and recombination rates. Distinct clusters fail to arise where there are high levels of recombination between strains, with no decline, or a slow decline, in the probability of recombination with increasing genetic distance. In this situation, subclusters appear to arise but become drawn back into the main population, presumably owing to the cohesive effect of recombination. In contrast, where recombination declines sharply with increasing genetic distance, separate clusters arise and then are maintained by distance-scaled recombination—high rates of recombination within each cluster and low rates between different clusters. In this scenario, clusters arise even though rates of recombination between similar isolates are substantially higher than those that prevent clusters from emerging in simulations where recombination rate is not scaled to genetic distance. These distinct clusters are effectively ‘species’, and the cohesive role of recombination within a cluster, and low rates between clusters, provides an attractive parallel with the biological species concept of Mayr (1942).

Empirical estimates of the relationship between genetic distance and recombination rate (*r*) are determined in terms of sequence divergence (*x*) and are of the form log(*r*)=*r*_0_−18*x* (Majewski & Cohan 1999*b*; Majewski *et al*. 2000), indicating a relatively slow decline as a function of genetic distance. Further work is required to relate this to the allelic model used here, but a simple argument can be used to see that the reduction in recombination rate with genetic distance (divergence) we have used to generate clusters is much sharper than this empirically determined one. By linearizing the binomial probability for sequence identity based on randomly scattered polymorphisms, we infer that *D*≈*Lx*, where *L* is the total length of sequence compared. Thus, the degree of sequence divergence should be very low, except when very few alleles match at all, and the degree of recombination should be nearly equal to that between identical sequences.

Under neutral drift, we therefore do not predict speciation in sympatric populations with high rates of recombination, unless the empirically determined reduction in recombination rate with sequence divergence is much steeper than that which has been reported. Based on the reported relationship (Majewski & Cohan 1999*b*; Majewski *et al*. 2000), we expect distance-scaled recombination to reinforce and maintain genetic separations which are initially created by allopatry or niche differentiation, but not to generate them.

In the absence of recombination, or when recombination is less frequent than mutation, a threshold is crossed and the population structure is instead effectively clonal, with clusters emerging as a consequence of divergence without bound (Cohan 2002) and stochastic loss of intermediate genotypes. These clusters are not produced by the same mechanism, and therefore they do not correspond to the same species definition as in the case of distance-scaled recombination.

The rates with which the processes modelled here would occur in nature are not clear. In the neutral simulations, we can observe the generation times at which resolved clusters appear, but generation length is a difficult concept for prokaryotes. Not only may rates of cell division vary widely depending on access to nutrients, but this may not even be the relevant generation time. In some circumstances, it may be appropriate to consider multiple bacteria as a single ‘soma’: for instance, in the case of a colony of bacteria in the throat of a host or a colony of yeast on the bark of a tree (Koufopanou *et al*. 2006). The generation time may then be better thought of as the time between the colonization of new hosts or sites.

Another characteristic of the clusters we observe is their dynamic nature. In all simulations, clusters emerge and become extinct, but there is considerable variation in the timespans involved. In these simulations, clusters compete with each other and result in stochastic extinctions, whereas in real populations ecological distinctiveness arising between clusters would be expected to have a large impact on their relative rates of extinction. One problem that arises from the observed dynamics is that any cross-sectional study of a real population is likely to identify clusters. There is no easy way of distinguishing whether two (or more) clusters observed within a sample of a natural population define clusters that are destined to remain distinct and which should each be given species designation, or are transient and destined to merge back into a single cluster, or are the consequence of inadequate sampling.

Recombination is believed to often involve the replacement of small regions (a few kilobases) of a recipient chromosome with the corresponding region from a donor strain. An important caveat of our simulations is that studies of natural populations, and the general features of RecA-mediated recombination, suggest that the rate of recombination will depend on the local sequence similarities between the donor and recipient strains in the regions involved in the localized genetic exchange. In this work, we assume that recombination is a function of overall genetic distance, measured as the proportion of the 140 alleles that differ between strains. We chose this as our initial approach, as computationally it allows an examination of large populations defined at large numbers of alleles. Further developments of our simulations incorporating local measures of genetic distance will be described elsewhere. Our observation of a recombination threshold, lying between *ρ*/*θ*=1 and *ρ*/*θ*=10 for situations where recombination is not distance-scaled, above which population cohesiveness is maintained and below which clonal clusters emerge, is not dependent on this assumption. Interestingly, Falush *et al*. (2006) present a simulation using local measures of genetic distance, which shows similar patterns of clustering and ‘speciation’, although we would argue that (as in our simulations) speciation at high recombination rates is only observed in their simulations under conditions where the recombination rate reduces with sequence divergence very much more steeply than found in empirical studies.

Bacteria exist as diverse populations that can be resolved into clusters which can be assigned to various taxonomic divisions (lineages, species, genera, etc.). The study of these populations and their taxonomy can be enhanced by the widespread application of multilocus sequencing approaches, with the prospect of future sequencing technologies making it ever more feasible to sequence hundreds of genes from thousands of strains. Within this context, it is important that we have some theoretical means of describing what we expect to observe in nature, of interpreting what we observe, and of integrating these findings with the biology and ecology of the organisms under study. Future work should address the impact of selection and population subdivision (or allopatry) on the nature and dynamics of genotypic clustering, as well as better models of genetic distance, and it should explicitly attempt to compare the clusters derived from simulation with those observed among natural populations.

## Supplementary Material

Fraser Suppl Movie 1 A population of 10^6^ bacteria was evolved for 10^6^ generation with high rates of recombination (*θ*=2, *ρ*=20), as in Figure 3. The clustering patterns are displayed using MDS for samples of 1000 isolates throughout the simulation. Individual frames from the movie are shown in Figure 3. In order to see movies, files should be opened in Windows Picture and Fax Viewer. The generation number is shown in the top left

Fraser Suppl Movie 2 As above. Except the movie shown corresponds to figure 4 of the main text where recombination rate is scaled to genetic distance
