# Supplementary figures and images for: Modelling bacterial speciation
Source: Philos Trans R Soc Lond B Biol Sci. 2006 Oct 6;361(1475):2039–44. doi: 10.1098/rstb.2006.1926 (PMC1764933; doi:10.1098/rstb.2006.1926)

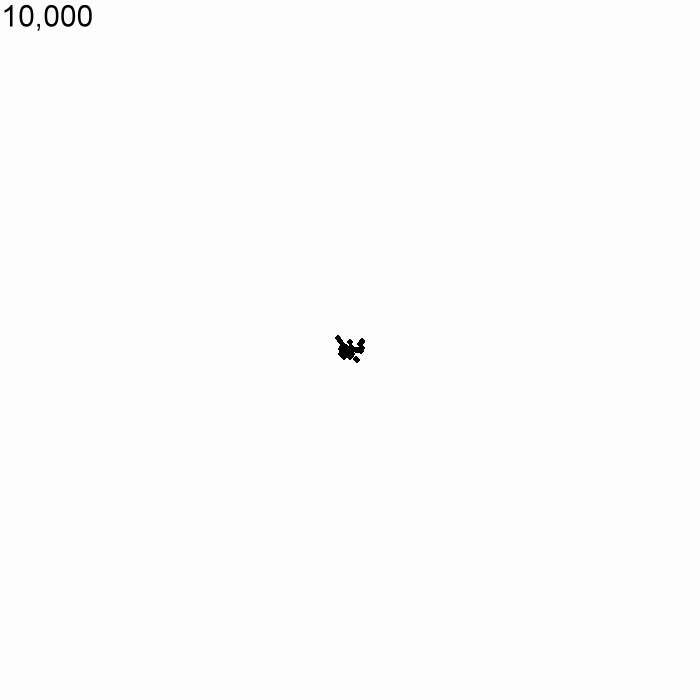

Supplement: Fraser Suppl Movie 1 — A population of 106 bacteria was evolved for 106 generation with high rates of recombination (θ=2, ρ=20), as in Figure 3. The clustering patterns are displayed using MDS for samples of 1000 isolates throughout the simulation. Individual frames from the movie are shown in Figure 3. In order to see movies, files should be opened in Windows Picture and Fax Viewer. The generation number is shown in the top left [file rstb20061926s02.gif]

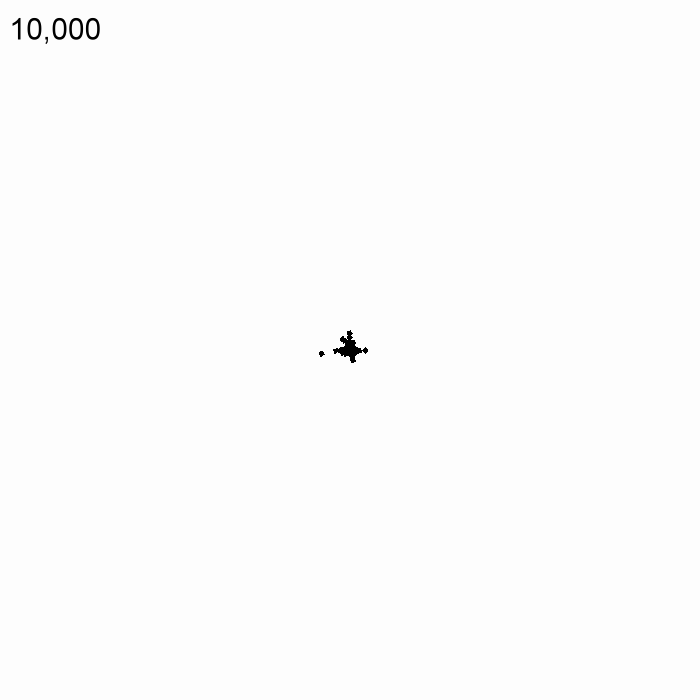

Supplement: Fraser Suppl Movie 2 — As above. Except the movie shown corresponds to figure 4 of the main text where recombination rate is scaled to genetic distance [file rstb20061926s03.gif]
